# Paediatric case of gastrointestinal basidiobolomycosis mimicking appendicitis – Case report

**DOI:** 10.1016/j.ijscr.2019.09.001

**Published:** 2019-09-18

**Authors:** Rami Issam Arabi, Abdullah Aljudaibi, Bashaer Ahmed Shafei, Hatoon Mohammed AlKholi, Maged Ezzat Salem, Khalid Ali Eibani

**Affiliations:** a Ibn Sina National College for Medical Studies, Jeddah, Saudi Arabia; b King Abdulaziz Hospital, Jeddah, Saudi Arabia

## Abstract

**Introduction:**

Basidiobolomycosis is a rare fungal infection caused by *Basidiobolus ranarum* that normally leads to subcutaneous infection and, rarely, to involvement in the gastrointestinal tract.

**Presentation of case:**

A 6-year-old boy presented to the Emergency department (ED) complaining of abdominal pain of a few weeks’ duration in the right lower quadrant, associated with fever and vomiting. A computed tomography (CT) of the abdomen was done. It showed an enlarged tubular structure in the right iliac fossa, a suspected appendicular lesion, and free fluid collection, as well as multiple enlarged mesenteric and right iliac lymph nodes. Based on these results, the decision was made to do surgical exploration on the patient. After two weeks, the patient developed tachycardia, abdominal distention, constipation, and spikes of fever. Two days later, re-exploration was performed. An ileostomy and colostomy were performed and drains inserted. Post-operative, the patient was pushed to paediatric intensive care unit (PICU), while still intubated and hemodynamically stable. However, he developed cardiac arrest on the same day and cardiopulmonary resuscitation (CPR) was performed, but he couldn't be resuscitated.

**Discussion:**

The patient presented with severe abdominal pain in the right lower quadrant, as well as fever and vomiting. These complaints may have been misdiagnosed as appendicitis. The diagnosis of gastrointestinal basidiobolomycosis was confirmed by histopathology, based on a surgical specimen taken during the primary exploration. The management was a combination between surgical intervention and medical treatment.

## Highlights

• Basidiobolomycosis is a rare fungal infection that leads to subcutaneous infection.
• Gastrointestinal basidiobolomycosis is difficult to diagnose primarily due to its non-specific clinical presentation.
• Gastrointestinal basidiobolomycosis should be a differential especially in paediatric patients present with abdominal mass and eosinophilia.
• Optimal way to manage gastrointestinal basidiobolomycosis is by surgical resection followed by 3 months of antifungal treatment.

## 1 Introduction

*Basidiobolus ranarum* (*B. ranarum*) is a fungus of zygomycetes class found in soil and the lung of reptiles, amphibians, and bats that can be transmitted through traumatic inoculation, which causes basidiobolomycosis, a rare fungal infection known to cause subcutaneous infection, with rare gastrointestinal manifestations [1,2]. Gastrointestinal basidiobolomycosis mostly involves the colon [1,3], and it's difficult to diagnose due to its non-specific presentation, as the patient may have abdominal pain, fever, constipation or diarrhea, and weight loss [[2], [3], [4]]. But it should be a differential, especially as paediatric patients who present with an abdominal mass and eosinophilia as gastrointestinal basidiobolomycosis can receive a misdiagnosis of appendicular mass, intestinal tuberculosis, or malignancy [5]. Basidiobolomycosis has been reported in both children and adults [2].

We report a case following the SCARE criteria of 6-year-olds [6], Saudi, male patient who presented to the ED with abdominal pain, especially in the right lower quadrant, fever, and vomiting. He was diagnosed with inflammatory bowel disease, secondary to intra-abdominal abscess, due to basidiobolomycosis. The hospital/legal team assumes full responsibility for this paper and ensures that all the patient information has been anonymized.

## 2 Case report

**Fig. 1:**
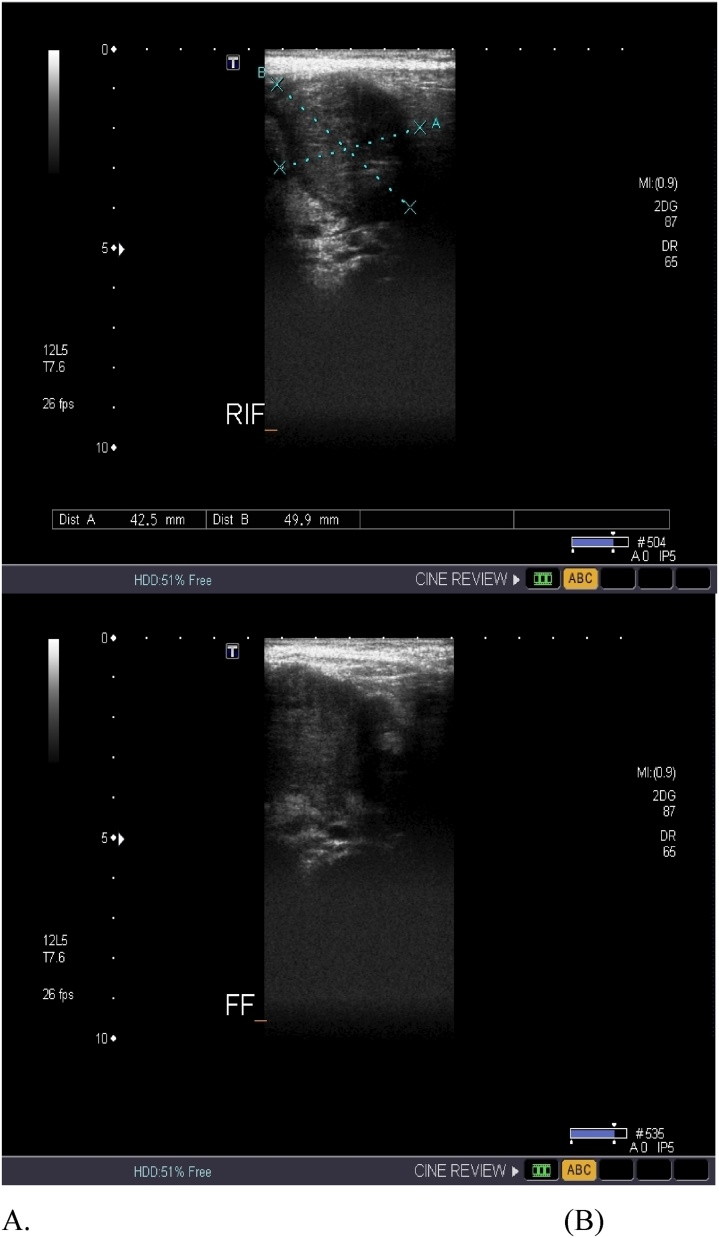
A) Abdominal ultrasound showing appendicular mass. B) Abdominal ultrasound showing fluid collection.

**Fig. 2:**
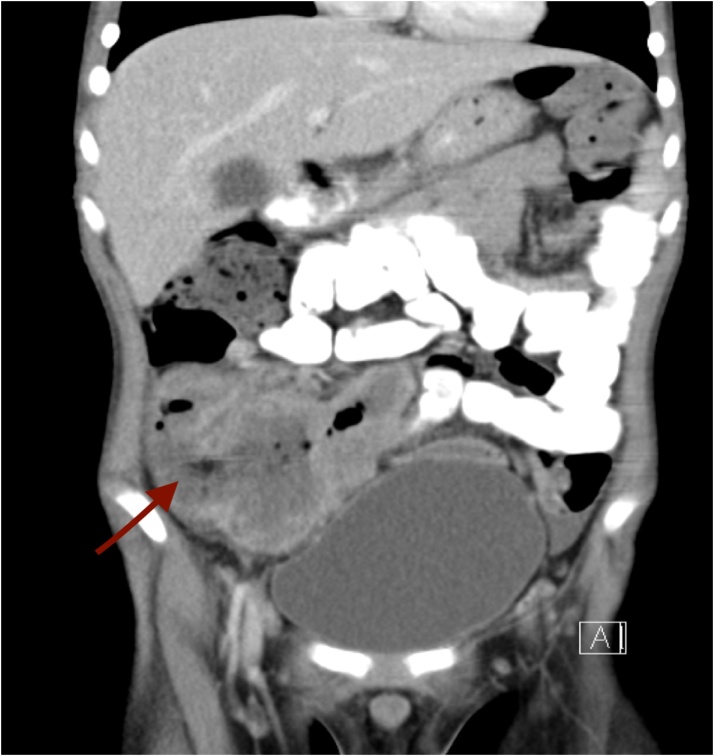
CT scan showing a large complex lesion in the right iliac fossa displacing the bladder and bowel loops medially.

**Fig. 3:**
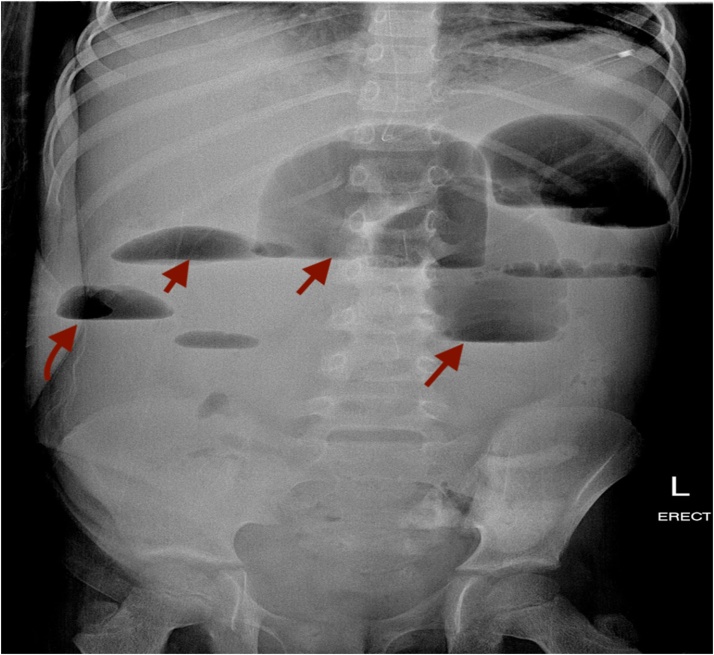
Post-operative abdominal X-ray showing multiple air fluid levels.

A previously healthy, immunocompetent 6-year-old boy presented to the Emergency room (ER) complaining of abdominal pain in the right lower quadrant that had progressed to be severe in the previous 10 days. The abdominal pain was associated with fever and vomiting, with no history of nausea, anorexia, change in bowel habits, or urinary symptoms. A few weeks prior to the hospital visit, the patient sought medical advice, and he was given IV antibiotics and discharged home on oral antibiotics for suspected complicated appendicitis, along with urinary tract infection. Upon physical examination, the patient was conscious and alert. His vital signs upon presentation: pulse 120 beats per minute; blood pressure 93/66 mmHg; and temperature 36.7 C. The abdomen was soft and lax, with tenderness in the right iliac fossa and a mass felt in the right lower abdomen. The initial laboratory workup revealed: white blood cells (WBCs) 20.4 k/uL; neutrophils (NE) 61.5%; haemoglobin (HB) 10.1 g/dL; and, sodium (Na) 132 mmol/L. Imaging studies, including abdominal ultrasound (US) and CT abdomen with oral and intravenous (IV) contrast, were done. Abdominal US showed hepatomegaly. A hypoechoic mass was seen at the right iliac fossa, measuring 4.9 cm × 4.2 cm, with fluid collection and an appendicular mass (Fig. 1). The CT abdomen showed: 1) enlarged tubular structures in the right iliac fossa region, measuring 8 cm × 2.5 cm, related to the cecum; 2) suspected appendicular lesions; 3) free fluid collection; and 4) multiple enlarged mesenteric and right iliac lymph nodes (Fig. 2). Based on these results, the decision was made to do a laparotomy exploration on the patient. Intra-operative findings revealed a large amount of pus, multiple intraperitoneal masses related to terminal ileum, paracecal and proximal ileal loops, and an inflamed mass at the paracecal region, with necrotic wall of cecum and appendix. An ileocecectomy and specimen for histopathology was sent, with two drains inserted. The post-operative course was smooth at the beginning, and the patient was extubated and shifted to the ward, where he received a 3rd generation of cephalosporins and metronidazole and was kept nil per os (NPO) on a nasogastric tube (NGT). Day 6 post-operative, an oral fluid diet was reintroduced gradually and was well tolerated, which led us to the resumption of a normal diet. Post-operative day 8, the result of histopathology revealed a large cecal ulcer (5 cm), with ileocecal mass with focal cecal perforation, wall thickening rich in eosinophils, and thick fungal hyphae Basidiobolomycosis. However, we didn’t start antifungal treatment because the patient was in stable condition. Afterwards, the patient started to have a surgical wound infection, with positive C/S of pseudomonas aeruginosa. Day 15 post-operative, he started to deteriorate with fever and vomiting. On the subsequent day, he developed tachycardia, abdominal distention, constipation, and spikes of fever. The patient was kept NPO, and NGT was inserted, which brought a moderate amount of brownish content. An abdominal X-ray, which was ordered to be done on a daily basis, showed multiple air fluid levels, indicating intestinal obstruction (Fig. 3). Emergent abdominal US revealed minimal fluid in the right iliac fossa, most likely postsurgical status. Blood workup results demonstrated WBCs 23.8 k/uL; NE 88.1%; HB 11.2 g/dL; and normal urea and electrolytes. On the next day, HB dropped to 9.6 g/dL, with persistent bilious gastric aspiration, abdominal distension, and tenderness. Post-operative day 18, the patient was pushed to the operation room for re-exploration. Intra-operative findings included multiple intra-abdominal abscesses eroding the small bowel with an inflamed bowel wall, multiple necrotic segments, and severe adhesions with inflammatory masses invading the rectosigmoid region. A small ileostomy and colostomy were performed and drains inserted. Post-operative, the patient was pushed to PICU while still intubated, sedated, and hemodynamically stable. Later, on the same day, 40 min of CPR was performed after he arrested, but he couldn't be resuscitated.

Connected to a tubular enhancing lesion likely matching the clinical diagnosis of appendicular abscess.

## 3 Discussion

Zygomycosis is a rare fungal infection caused by two forms: Mucormycosis and Entomophthoromycosis [1]. *Conidiobolus coronatus* and *B. ranarum* are the two major species causing entomophthoromycosis [2,5]. The first isolation of *B. ranarum* was in 1955 from crumbling plants [7]. The first recorded human infection caused by *B. ranarum* was in 1956. [8] Gastrointestinal basidiobolomycosis was diagnosed for the first time in 1980 in a 4-year-old child that died soon after the diagnosis due to a disseminated fungal infection [4], and the first culture proving gastrointestinal basidiobolomycosis was reported in 1986 in the United States [9]. Basidiobolomycosis is known to cause soft tissue infections, especially in tropical areas [4,5]. Recently, an increase in the incidence of basidiobolomycosis infection has been reported, which may be attributed to the increase in the immunocompromised state of general populations around the world [4]. The risk factors for basidiobolomycosis remain unclear, and it's not obvious how the fungus get inside the gastrointestinal tract of the patients in cases of gastrointestinal basidiobolomycosis. However, it's suspected that ingestion of food contaminated by soil or animal faeces is the source [3]. In our case, the patient presented with severe abdominal pain in the right lower quadrant, fever, and vomiting, and these complaints may be misdiagnosed as appendicitis, or inflammatory bowel disease. For this reason, the diagnosis of gastrointestinal basidiobolomycosis poses some difficulties. The diagnosis of basidiobolomycosis infection was made based on a surgical specimen. The management was a combination of surgical intervention and medical treatment. Based on literature, the optimal way to manage gastrointestinal basidiobolomycosis is by surgical resection of all necrotic bowel and debridement for any involved tissue, followed by three months of antifungal treatment [3]. The best antifungal agent remains debatable, with previously reported treatment failure with amphotericin B [9,10], but with the use of itraconazole considered an effective choice in gastrointestinal basidiobolomycosis [9,11,12].

## 4 Conclusion

Gastrointestinal basidiobolomycosis is difficult to diagnose primarily due to its non-specific clinical presentation in which it can mimic appendicitis or malignancy. These cases are prone to misdiagnosis. The final diagnosis can be confirmed only by sending the lesion for histopathology, fungal cultures and molecular testing for basidiobolomycosis. This case report describes a child with severe gastrointestinal basidiobolomycosis in which it leads to fatality.

## Sources of funding

No sources of funding for our paper.

## Ethical approval

Ethical approval has been taken from the ministry of health.

## Consent

Unfortunately, the patient deceased and we could not establish any sort of communication with the patient. However, the hospital/legal team assume full responsibility for this paper and ensure that all the patient information has been anonymized. A copy of the letter is available for review by the Editor-in-Chief of this journal on request.

## Author’s contribution

Rami Arabi MD: wrote the introduction co-wrote the discussion reviewed the manuscript critically.

Abdullah Aljudaibi MD: Co-wrote the discussion part reviewed the manuscript critically.

Bashaer Ahmed Shafei, MD: Co-wrote the case presentation part.

Hatoon Mohammed AlKholi, MD: Co-wrote the case presentation part.

Maged Ezzat Salem, MD: reviewed the manuscript critically.

Khalid Ali Eibani, MD: Final approval of the manuscript.

## Registration of research studies

Not applicable.

## Guarantor

Bashaer Ahmed Shafei, MD.

## Provenance and peer review

Not commissioned, externally peer-reviewed.

## Declaration of Competing Interest

No conflict of interest between authors.
